# Quantification of longitudinal tissue *p*O_2_ gradients in window chamber tumours: impact on tumour hypoxia

**DOI:** 10.1038/sj.bjc.6690273

**Published:** 1999-04

**Authors:** M W Dewhirst, E T Ong, R D Braun, B Smith, B Klitzman, S M Evans, D Wilson

**Affiliations:** Departments of Radiation OncologyRadiologySurgery, Duke University Medical Center, Box 3455, Durham, 27710, NC; Departments of Radiation Oncology andBiochemistry and Biophysics, University of Pennsylvania, Philadelphia, PA, USA

**Keywords:** hypoxia, oxygen transport, tumours, longitudinal gradients

## Abstract

We previously reported that the arteriolar input in window chamber tumours is limited in number and is constrained to enter the tumour from one surface, and that the *p*O_2_ of tumour arterioles is lower than in comparable arterioles of normal tissues. On average, the vascular *p*O_2_ in vessels of the upper surface of these tumours is lower than the *p*O_2_ of vessels on the fascial side, suggesting that there may be steep vascular longitudinal gradients (defined as the decline in vascular *p*O_2_ along the afferent path of blood flow) that contribute to vascular hypoxia on the upper surface of the tumours. However, we have not previously measured tissue *p*O_2_ on both surfaces of these chambers in the same tumour. In this report, we investigated the hypothesis that the anatomical constraint of arteriolar supply from one side of the tumour results in longitudinal gradients in *p*O_2_ sufficient in magnitude to create vascular hypoxia in tumours grown in dorsal flap window chambers. Fischer-344 rats had dorsal flap window chambers implanted in the skin fold with simultaneous transplantation of the R3230AC tumour. Tumours were studied at 9–11 days after transplantation, at a diameter of 3–4 mm; the tissue thickness was 200 μm. For magnetic resonance microscopic imaging, gadolinium DTPA bovine serum albumin (BSA-DTPA-Gd) complex was injected i.v., followed by fixation in 10% formalin and removal from the animal. The sample was imaged at 9.4 T, yielding voxel sizes of 40 μm. Intravital microscopy was used to visualize the position and number of arterioles entering window chamber tumour preparations. Phosphorescence life time imaging (PLI) was used to measure vascular *p*O_2_. Blue and green light excitations of the upper and lower surfaces of window chambers were made (penetration depth of light ~50 vs >200 μm respectively). Arteriolar input into window chamber tumours was limited to 1 or 2 vessels, and appeared to be constrained to the fascial surface upon which the tumour grows. PLI of the tumour surface indicated greater hypoxia with blue compared with green light excitation (*P* < 0.03 for 10th and 25th percentiles and for per cent pixels < 10 mmHg). In contrast, illumination of the fascial surface with blue light indicated less hypoxia compared with illumination of the tumour surface (*P* < 0.05 for 10th and 25th percentiles and for per cent pixels < 10 mmHg). There was no significant difference in *p*O_2_ distributions for blue and green light excitation from the fascial surface nor for green light excitation when viewed from either surface. The PLI data demonstrates that the upper surface of the tumour is more hypoxic because blue light excitation yields lower *p*O_2_ values than green light excitation. This is further verified in the subset of chambers in which blue light excitation of the fascial surface showed higher *p*O_2_ distributions compared with the tumour surface. These results suggest that there are steep longitudinal gradients in vascular *p*O_2_ in this tumour model that are created by the limited number and orientation of the arterioles. This contributes to tumour hypoxia. Arteriolar supply is often limited in other tumours as well, suggesting that this may represent another cause for tumour hypoxia. This report is the first direct demonstration that longitudinal oxygen gradients actually lead to hypoxia in tumours. © 1999 Cancer Research Campaign


*British Journal of Cancer* (1999) **79**(11/12), 1717–1722
© 1999 Cancer Research Campaign
Article no. bjoc.1998.0273

### Quantification of longitudinal tissue $pO_2$ gradients in window chamber tumours: impact on tumour hypoxia

**MW Dewhirst[1], ET Ong[1], RD Braun[1], B Smith[2], B Klitzman[3], SM Evans[4] and D Wilson[5]**

Departments of [1]Radiation Oncology, [2]Radiology and [3]Surgery, Box 3455, Duke University Medical Center, Durham, NC 27710; Departments of [4]Radiation Oncology and [5]Biochemistry and Biophysics, University of Pennsylvania, Philadelphia, PA, USA

**Summary** We previously reported that the arteriolar input in window chamber tumours is limited in number and is constrained to enter the tumour from one surface, and that the $pO_2$ of tumour arterioles is lower than in comparable arterioles of normal tissues. On average, the vascular $pO_2$ in vessels of the upper surface of these tumours is lower than the $pO_2$ of vessels on the fascial side, suggesting that there may be steep vascular longitudinal gradients (defined as the decline in vascular $pO_2$ along the afferent path of blood flow) that contribute to vascular hypoxia on the upper surface of the tumours. However, we have not previously measured tissue $pO_2$ on both surfaces of these chambers in the same tumour. In this report, we investigated the hypothesis that the anatomical constraint of arteriolar supply from one side of the tumour results in longitudinal gradients in $pO_2$ sufficient in magnitude to create vascular hypoxia in tumours grown in dorsal flap window chambers. Fischer-344 rats had dorsal flap window chambers implanted in the skin fold with simultaneous transplantation of the R3230AC tumour. Tumours were studied at 9–11 days after transplantation, at a diameter of 3–4 mm; the tissue thickness was 200 μm. For magnetic resonance microscopic imaging, gadolinium DTPA bovine serum albumin (BSA-DTPA-Gd) complex was injected i.v., followed by fixation in 10% formalin and removal from the animal. The sample was imaged at 9.4 T, yielding voxel sizes of 40 μm. Intravital microscopy was used to visualize the position and number of arterioles entering window chamber tumour preparations. Phosphorescence life time imaging (PLI) was used to measure vascular $pO_2$. Blue and green light excitations of the upper and lower surfaces of window chambers were made (penetration depth of light ~50 vs >200 μm respectively). Arteriolar input into window chamber tumours was limited to 1 or 2 vessels, and appeared to be constrained to the fascial surface upon which the tumour grows. PLI of the tumour surface indicated greater hypoxia with blue compared with green light excitation ($P < 0.03$ for 10th and 25th percentiles and for per cent pixels < 10 mmHg). In contrast, illumination of the fascial surface with blue light indicated less hypoxia compared with illumination of the tumour surface ($P < 0.05$ for 10th and 25th percentiles and for per cent pixels < 10 mmHg). There was no significant difference in $pO_2$ distributions for blue and green light excitation from the fascial surface nor for green light excitation when viewed from either surface. The PLI data demonstrates that the upper surface of the tumour is more hypoxic because blue light excitation yields lower $pO_2$ values than green light excitation. This is further verified in the subset of chambers in which blue light excitation of the fascial surface showed higher $pO_2$ distributions compared with the tumour surface. These results suggest that there are steep longitudinal gradients in vascular $pO_2$ in this tumour model that are created by the limited number and orientation of the arterioles. This contributes to tumour hypoxia. Arteriolar supply is often limited in other tumours as well, suggesting that this may represent another cause for tumour hypoxia. This report is the first direct demonstration that longitudinal oxygen gradients actually lead to hypoxia in tumours.

**Keywords:** hypoxia; oxygen transport; tumours; longitudinal gradients

Classically, the origin of chronic tumour hypoxia has been attributed to radial gradients of oxygen, which is the decline in oxygen tension as the distance into the tissue increases away from the vessel (Thomlinson and Gray, 1955). Although it has been suspected that such gradients existed, based on the common observation of necrosis at defined distances from vessels and patterns of hypoxia marker uptake in tumours (Evans et al, 1995; Raleigh et al, 1996), direct measurements of radial oxygen tension gradients have only recently been made. The existence of radial gradients has been verified recently in window chamber tumours using polarographic electrodes as well as with phosphorescence lifetime imaging (PLI) (Dewhirst et al, 1994; Helmlinger et al, 1997). In both studies, however, it was also noted that tumour vascular $pO_2$ was often quite low and, in such cases, the radial gradient was virtually zero because the source vessel contained little or no oxygen. In a previous study, we found that 25% of functional vessels had oxygen tensions that were indistinguishable from zero (Dewhirst et al, 1992*a*). An important question to answer is why intravascular hypoxia exists in tumours.

We have previously reported that tumours grown in window chambers have an arteriolar supply that is limited to the fascial surface of the chamber (Dewhirst et al, 1996*a*), and thus suspected that a longitudinal tissue $pO_2$ gradient might, in part, provide an explanation for intravascular hypoxia on the upper tumour surface. Using PLI, we sought to investigate this question in tumour-bearing window chambers of Fischer-344 rats.

*Received 1 April 1998*
*Accepted 24 July 1998*

*Correspondence to:* MW Dewhirst

### MATERIALS AND METHODS

### Animal model

Fischer-344 rats (100–150 g, Charles River Laboratories, Raleigh, NC, USA) were surgically implanted with cutaneous window chambers as described previously (Papenfuss et al, 1979). R3230AC

**Table 1** Comparison of oxygen tension distributions on tumour and fascial surfaces of window chamber tumours using blue and green light excitation

| Excitation wavelength | $n$ | Surface | Median | $pO_2$ (mmHg) [mean (s.e.m.)] 10th percentile | 25th percentile |
|---|---|---|---|---|---|
| Blue | 16 | Tumour | 25 (4) | 11 (2) | 16 (2) |
| Green | 16 | Tumour | 32 (4) | 15 (2)[a] | 21 (3)[a] |
| Blue | 6[b] | Tumour | 19 (6) | 8 (3) | 11 (4) |
| Green | 6[b] | Tumour | 25 (5) | 10 (1) | 15 (3) |
| Blue | 6[b] | Fascial | 40 (7) | 21 (5)[c] | 28 (6)[c] |
| Green | 6[b] | Fascial | 26 (4) | 12 (2) | 17 (3) |

[a]Indicates significant difference between blue and green light excitation of the same surface, $P < 0.03$. [b]Indicates a subset of the total set of 16, in which both tumour and fascial surfaces were analysed in the same individuals. [c]Indicates significant difference between the tumour and fascial surfaces under the same light excitation, $P < 0.05$.

mammary adenocarcinomas were transplanted onto a fascial plain of subcutaneous tissue at the time of window chamber surgery. After surgery, but before experimentation, the animals were housed individually in an environmental chamber maintained at 34°C and 50% humidity with continuous access to food and water. Imaging studies were performed 9–11 days after transplantation. The maximum tissue thickness was 200 μm. The surgical preparation and housing of chamber-bearing rats was performed at DUMC (Duke University Medical Center). Magnetic resonance imaging and intravital microscopy procedures were performed at DUMC. PLI studies were performed at the University of Pennsylvania. After implantation of the window chambers and tumour growth, the animals were shipped to the University of Pennsylvania within 2 days of PLI studies. All protocols were approved by the DUMC Institutional Animal Care and Use Committee.

### Anaesthesia

Animals were anaesthetized with sodium pentobarbital (50 mg $kg^{-1}$ i.p.) for all surgical and experimental procedures. Body temperature was maintained using Deltaphase Isothermal pads (Model 39 DP, Braintree Scientific, Braintree, MA, USA). Blood pressures were monitored using a digital manometer (FiberOptic Sensor Technologies, Ann Arbor, MI, USA) and averaged 90–100 mmHg, which is typical for this anaesthetic regimen in our hands (Dewhirst et al, 1996*a*; Shan et al, 1997) (data not shown).

### Intravital microscopy

In a separate group of animals, visualization of the vasculature of both tumour surfaces was performed using intravital microscopy (Zeiss MPS Intravital Microscopy System, Zeiss, Thornwood, NY, USA). The window chamber tissue was imaged at 25× and images were recorded using a colour 3CCD video camera (model ZVS 3C75DE, Optronics Engineering, Goleta, CA, USA) and frames were captured with a frame grabber (model LG3PCIRS170, Scion Corporation, Frederick, MD, USA) installed on a microcomputer (model G6-233, Gateway 2000, Kansas City, MO, USA). These images were subsequently analysed using image analysis software (NIH Image).

### Magnetic resonance microscopy

In a separate group of animals, three-dimensional visualization of tumour vasculature was obtained using magnetic resonance microscopy, with Gd-albumin administered as a contrast agent (Smith et al, 1994). Briefly, the upper glass window of the tumour chamber was removed from the anaesthetized rat (40–50 mg $kg^{-1}$, i.p. nembutal) and the skin flap was covered with phosphate-buffered saline (PBS). One millilitre of bovine serum albumin diethylenetriaminepentaacetic anhydride-gadolinium (BSA-DTPA-Gd) with approximately 1 mM Gd was injected i.v. Fifteen seconds after administration of the contrast agent, the skin flap was immersed in 10% formalin and surgically removed from the animal. The tumour sample was embedded in 3% agarose and placed in a 1-cm solenoid imaging coil. Imaging was performed on a 9.4-T Bruker Instruments magnet with an Omega System console (Freemont, CA, USA). Data were acquired using three-dimensional spin warp encoding adapted for imaging large arrays. The array size by pixels was $256^3$, with pixels of 40 μm on a side [repetition time (TR) = 200 ms, echo time (TE) = 6 ms], and four excitations for each phase-encoding step.

### Phosphorescence lifetime imaging (PLI)

PLI was used to measure vascular $pO_2$, after i.v. administration of 3.5 mg Pd-mesotetra-(4-carboxyphenyl) porphyrin (oxyphore). Blue (419 nm) and green light (525 nm) excitations (BLE and GLE respectively) from the upper and lower tumour surfaces were made. The porphyrin has absorption peaks at both of these wavelengths, but the emitted phosphorescence spectrum and lifetime are independent of the wavelength of excitation. Phosphorescence was imaged using a grated, intensified CCD camera. The camera was turned on at varying times after the flash of excitation light. The rationale for using both wavelengths is that the penetration depth for blue light (approximately 50 μm) will be less than that for green light (>200 μm) because of differences in absorption by light absorbing chromophores, such as cytochromes haemoglobin and myoglobin. Details of these methods have been published previously (Wilson et al, 1992; Vinogradov et al, 1996; Cerniglia et al, 1997).

The animals were anaesthetized, oxyphore was administered i.v. via the tail vein and imaging commenced within 5–10 min of injection. The total imaging period lasted no more than 30 min, after which time the animal was killed using an overdose of nembutal. The size of the oxyphore complex is large and most of the signal observed is intravascular during this period of observation (Wilson et al, 1992). In the first ten experiments, a direct comparison between green and blue light excitation was made on the tumour surface of the chamber only. After it was seen that

 © Cancer Research Campaign 1999

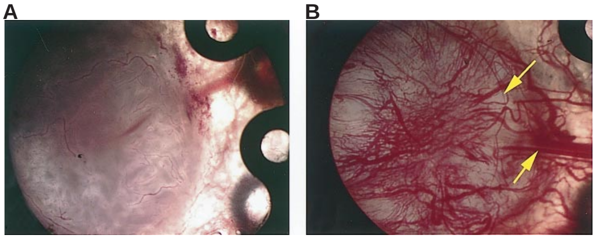

**Figure 1** Appearance of microvasculature on tumour (**A**) and fascial (**B**) surfaces of a window chamber preparation. On the fascial surface, two arterioles are readily visible, as indicated by the arrows. The larger of the two has visible smooth muscle and is seen diving into the tumour parenchyma (lower arrow). Its shadow is seen on the tumour surface (**A**), where it has penetrated into the tissue. A smaller arteriole is also seen with small branches entering the tumour tissue (upper arrow)

there were substantial differences for this comparison, an additional six animals were studied in which blue and green light excitation of both the tumour and fascial surfaces was made to further characterize the nature of the gradients observed.

### Statistical methods

Differences in oxygenation parameters obtained using different excitation wavelengths (BLE vs GLE) or obtained from different surfaces of the window preparation (tumor vs fascial) were compared using the paired two-tail Student's *t*-test. Differences were considered significant when $P < 0.05$; all data are reported as means; standard errors of the mean (s.e.m.) are indicated in parentheses.

### RESULTS

### Orientation of tumour arterioles

The vascular structure of these tumours is quite different when visualizing it from either the fascial surface or the tumour surface (Figure 1). On the tumour surface, the vasculature is composed of predominantly tortuous venular structures of low overall vascular density. In contrast, the fascial surface has a luxuriant growth of vasculature with arterioles that are readily visible. Characteristics of arterioles that distinguished them from other microvessels were: (1) relatively straight course, with divergent flow; (2) visible evidence for muscular wall; and (3) divergent branch angles that are near 90°. These arterioles can be observed to traverse along the fascial surface beneath the tumour. Typically, one or two of these vessels will enter the tumour parenchyma and at the point of entry visual evidence of smooth muscle was frequently lost.

The magnetic resonance microscopic images substantiated the orientation of tumour-feeding arterioles (Figure 2). The vasculature of the fascial layer was quite dense. Large venules and/or arterioles with diameters greater than 40 µm (pixel size of images) were readily observed in these vascular networks. The upper surface of the tumour also showed a dense network of vessels, but the diameters were primarily less than or equal to 40 µm. In cross section, it was readily apparent that the vascular density in the interior of the tumour was less than that on either surface. However, microvessels with diameters >40 µm could be visualized penetrating through this space. Three tumours were studied in this way, but only one is shown for illustrative purposes because the others looked quite similar.

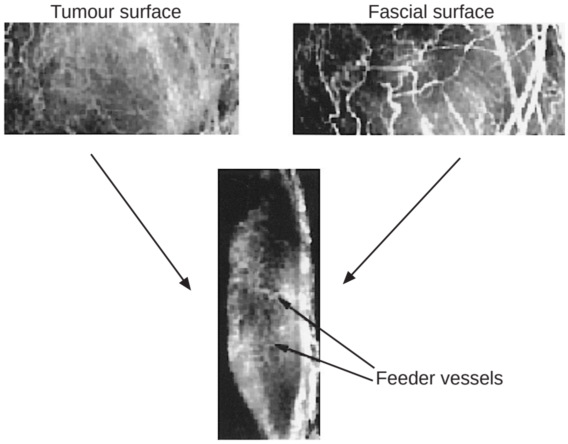

**Figure 2** Three-dimensional visualization of vasculature in a window chamber tumour. Data obtained using magnetic resonance microscopy as described in the text. The vascular density of the tumour and fascial surfaces is relatively high compared with the interior of the tumour. The path of vessels entering the tumour from feeding arterioles is indicated by the arrows. The pixel dimension for this image was 40 µm, limiting clear visualization of vessels with smaller diameters

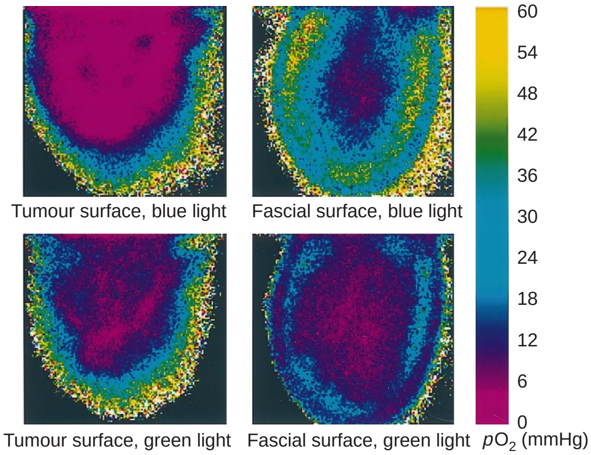

**Figure 3** Phosphorescence lifetime imaging of the tumour and fascial surfaces of a window chamber tumour, as elucidated by blue vs. green light excitation (BLE vs GLE respectively). In this example, the colour scale is set to visualize the entire $pO_2$ range. The colour bar indicates corresponding $pO_2$ values in mmHg. With BLE, the tumour surface appears more hypoxic compared with BLE of the fascial surface or GLE of either surface. This difference is attributed to the limited depth of penetration of blue light

### Phosphorescence lifetime imaging

Visualization of tissue $pO_2$ distribution can be set so that the entire range of values can be seen in the same image. When this has been carried out, gradients in $pO_2$, from the normal tissue periphery to the interior of the tumour, are easily visualized (Figure 3). With blue light excitation (BLE), the nadir of these gradients was greater in relative size when visualized from the tumour surface as opposed to the fascial surface. In the example shown, the tumour surface appears to be more hypoxic with BLE than GLE. In contrast, BLE of the fascial surface shows less hypoxia than when the same preparation is illuminated with green light. If the colour range for $pO_2$ is restricted to emphasize the range from the periphery to the centre of the tumour, then intratumoral heterogeneity in $pO_2$ distribution is more easily seen (Figure 4).

*© Cancer Research Campaign 1999*

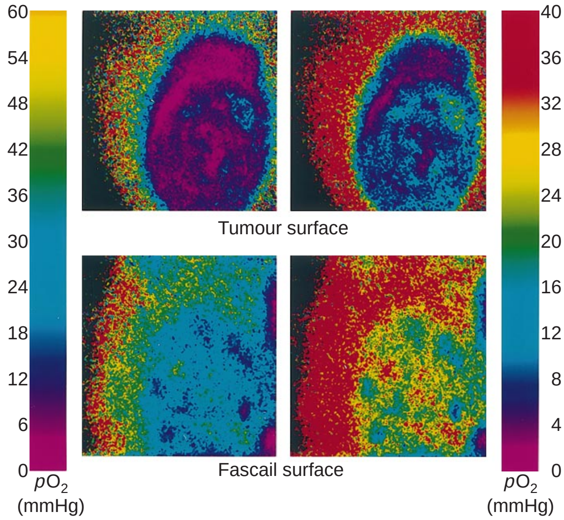

**Figure 4** Phosphorescence lifetime imaging of the tumour surface with thresholding to compare the entire $pO_2$ distribution (left column) vs. restriction to the range within the tumour (right column). Spatial heterogeneities in tumour surface $pO_2$ are more easily seen when the range is limited to $pO_2$ values between 0 and 40 mmHg

The average median $pO_2$ of the 16 tumours that were imaged from the tumour surface was 25 (±4) mmHg when measured with BLE and 32 (±4) mmHg when measured using GLE (Table 1). This difference was not statistically significant. However, there were significant differences when various parameters indicative of the lower end of the $pO_2$ distribution were compared. There were statistically significant differences in the 10th and 25th percentiles and the per cent of pixels <10 mmHg when comparing BLE and GLE ($P < 0.03$) with BLE, indicating a greater level of hypoxia than GLE. For example, the average percent of pixels < 10 mmHg was 22.1 (±5.6) for BLE vs 9.7 (±3.2) for GLE.

The average median $pO_2$ of the fascial surface was 40 (±7) mmHg when measured with BLE and 26 (±4) mmHg when measured with GLE. For all of the parameters studied, there was a trend towards a greater level of oxygenation with BLE than with GLE, but none of the comparisons were statistically significant. In the six window chambers where both surfaces were imaged, the gradient was observed more clearly. For example, with BLE the percent pixels <10 mmHg averaged 36.3 (±8.9) from the tumour surface vs. 6.9 (±4.0) when imaged from the fascial surface ($P < 0.05$). Similar differences were seen for the 10th and 25th percentiles of the frequency distribution. In contrast, the more deeply penetrating GLE did not show statistically significant differences when comparing tumour and fascial surfaces in the same six preparations.

### DISCUSSION

These data clearly show that there is a longitudinal gradient in tissue oxygenation in the window chamber tumour model that is set up by the geometric orientation of arterioles entering the tumour from one surface. The existence of the gradient was demonstrated because of differences in the depth of penetration of blue and green light into the window chamber tissue. When BLE of the tumour surface was used, the tissue appeared more hypoxic than with GLE because a thinner region of the tissue at the upper surface was being sampled with BLE. GLE penetrates completely through the tissue, thus providing signals from the whole thickness of the window. Similarly, in the subset of tumours in which both surfaces were illuminated with BLE, the tumour surface consistently appeared more hypoxic than the fascial surface.

It has been suspected for some time that the relative lack of arteriolar input into tumours may be a contributing cause to tumour hypoxia, but this study is the first to prove that limited arteriolar supply contributes to longitudinal gradients that result in tumour hypoxia. The relative lack of arteriolar supply to tumours has been discussed by a number of authors. For example, Lindgren and Docent (1945) performed extensive histological analysis of benign and malignant human tumours and found relatively little evidence for vascular smooth muscle in tumour vessels of either classification. Similar observations were made by Lagergran et al (1960) in examining a series of human sarcomas. These authors also performed angiography and noted the relative lack of arteriolar input to the tumours (Lagergren et al, 1960). Many other similar studies have been made in murine tumours, as reviewed by Warren (1979). Perhaps the most detailed analysis of afferent blood supply of murine tumours was made by Falk (1978, 1980). The three-dimensional orientation of vessels in tumours was recreated using a benzidine staining procedure of serial histological sections. With this method, he was able to trace the path of arterioles from the surrounding normal tissues and described the same type of afferent patterns that we describe herein.

In many normal tissues, there is a redundancy in arteriolar supply, or at least a repeated unit design in which the tissue volume supplied by a single arteriole is tightly regulated (e.g. the kidney). The relative number of arteriolar vessels is high enough that some authors have suggested that a major source for oxygen transport in some tissues is the arterioles, rather than the capillaries (Ellsworth and Pittman, 1990; Kerger et al, 1995; Torres et al, 1996). Longitudinal gradients also exist in normal tissues, but, because of the redundancy of vascular supply, such gradients do not lead to vascular or tissue hypoxia in most cases (Duling and Berne, 1970; Ellsworth et al, 1987; Swain and Pittman, 1989). The evidence from this paper and many others suggests that the volume of tissue supplied by a single artery in tumours can be much greater than for normal tissues. The anatomical constraints of a limited arteriolar supply being constrained to the periphery of the tumour is what allows for the steep longitudinal gradient leading to vascular and tissue hypoxia, as we have described in this paper.

The most frequently cited reason for chronic hypoxia in tumours is the presence of long intervascular distances (Thomlinson and Gray, 1955). We began to suspect that the origins of chronic hypoxia were more complex when we measured intravascular $pO_2$ in tumour vessels of the tumour surface of window chamber tumours, using recessed tip microelectrodes. Those results showed that these microvessels had perivascular $pO_2$ values averaging around 10 mmHg near the centre of the tumour mass (Dewhirst et al, 1992*a*). Twenty-five per cent of such vessels had $pO_2$ values below the level of detection (1 mmHg), in spite of the fact that they had blood flowing through them. Since that time, others have found corroborating evidence for the existence of hypoxia in functional tumour vessels. For example, Helmlinger et al (1997) recently demonstrated vascular hypoxia in window chamber tumours using the phosphorescence-quenching method. Fenton and Boyce (1994) used a combination of the freely diffusible vascular marker, Hoechst 33342 and cryospectrophotometry of haemoglobin in red cells of tumour vessels in tumours transplanted to the flank. A subset of vessels contained dye staining in the tissue immediately adjacent to the vessel as well

 *© Cancer Research Campaign 1999*

as haemoglobin saturation measurements indicative of hypoxia (Fenton and Boyce, 1994). The authors suggested that this may be due to transient vascular stasis between the time that the dye was given and tissue removal for cryospectrophotometry. However, an equally plausible explanation is that the vessels were functionally hypoxic, even though they had active flow and contained red cells.

However, as yet, there has been no conclusive proof that vessels with blood flow are hypoxic in spontaneous tumours. Immunohistochemical studies of patterns of tumour hypoxia, using hypoxia marker drugs, in canine and human tumours have typically reported that most intense binding occurs near regions of necrosis, which are typically farthest removed from the nearest microvessel (Cline et al, 1990; Kennedy et al, 1997). Immunohistochemical localization of microvessels would help to clarify this issue further because it is frequently difficult to positively identify microvessels on the basis of H and E staining alone. Alternatively, cryospectrophotometry studies in human tumours have demonstrated ample evidence for vascular hypoxia, but it was not known if such vessels had active blood flow at the time the tissue was removed (Mueller–Klieser et al, 1981).

Finally, it is of interest to compare the $pO_2$ values obtained in this study with our prior microelectrode studies with this same tumour model. We previously found that the overall average $pO_2$ of tumour vessels was near 20 mmHg (Dewhirst et al, 1992*a*), similar to what was observed in this study using the phosphorescence quench imaging method with BLE. In addition, the average $pO_2$ of tumour arterioles was over 32 mmHg (Dewhirst et al, 1996*a*), which is similar to the average $pO_2$ of the fascial surface of these preparations in the current study using BLE. Finally, we observed a $pO_2$ gradient when comparing the normal tissue surrounding the tumour to its periphery and centre. Similar results were shown in the current study. The fact that these two independent methods of oxygenation measurement provide similar results makes the case stronger that the magnitude of the measurements reflects the true pathophysiological state. It is becoming increasingly clear that chronic hypoxia comes from several sources. Although large intervascular distances play a role, as was described by Thomlinson and Gray (1955), there are other features of tumour microvascular function that may contribute. These include arteriolar $pO_2$ deoxygenation (Dewhirst et al, 1996*a*), plasma channels (Dewhirst et al, 1996*b*), rheological effects (Dewhirst et al, 1992*b*), irregularities in tumour vascular geometry (Secomb et al, 1993) and oxygen consumption rates that are out of balance with the ability of the vasculature to deliver oxygen (Secomb et al, 1995). In this paper, we have added another feature to this complex set of circumstances that may contribute to chronic hypoxia in tumours. The features of tumour angiogenesis that are not permissive for arteriolar ingrowth into the tumour create steep longitudinal oxygen gradients, which contribute to intravascular and tissue hypoxia.

### ACKNOWLEDGEMENTS

This work was supported by grants from the NIH/NCI, CA40355, P4105959, NS-31465 and CA56679. Thanks go to Ms Tina Jones for assistance in preparation of the manuscript and to Dr Chuan Li for assistance with the intravital microscopy.

### REFERENCES

Cerniglia GJ, Wilson DF, Pawlowski M, Vinogradov S and Biaglow J (1997) Intravascular oxygen distribution in subcutaneous 9L tumors and radiation sensitivity. *J Appl Physiol* **82**: 1939–1945

Cline JM, Thrall DE, Page RL, Franko AJ and Raleigh JA (1990) Immunohistochemical detection of a hypoxia marker in spontaneous canine tumors. *Br J Cancer* **62**: 925–931

Dewhirst MW, Ong ET, Klitzman B, Secomb TW, Vinuya RZ, Dodge R, Brizel D and Gross JF (1992*a*) Perivascular oxygen tensions in a transplantable mammary tumor growing in a dorsal flap window chamber. *Radiat Res* **130**: 171–182

Dewhirst MW, Ong ET, Madwed D, Klitzman B, Secomb T, Brizel D, Bonaventura J, Fosner G, Kavanagh B, Edwards J and Gross JF (1992*b*) Effects of the calcium channel blocker flunarizine on the hemodynamics and oxygenation of tumor microvasculature. *Radiat Res* **132**: 61–68

Dewhirst MW, Secomb TW, Ong ET, Hsu R and Gross JF (1994) Determination of local oxygen consumption rates in tumors. *Cancer Res* **54**: 3333–3336

Dewhirst MW, Ong ET, Rosner GL, Rehmus SW, Shan S, Braun RD, Brizel DM and Secomb TW (1996*a*) Arteriolar oxygenation in tumour and subcutaneous arterioles: effects of inspired air oxygen content. *Br J Cancer* (suppl. XXVII) **74**: S241–S246

Dewhirst MW, Kimura H, Rehmus SWE, Braun RD, Papahadjopoulos D, Hong K and Secomb TW (1996*b*) Microvascular studies on the origins of perfusion-limited hypoxia. *Br J Cancer* **74**: S247–S251

Duling B and Berne RM (1970) Longitudinal gradients in periarteriolar oxygen tension. *Circ Res* **27**: 669–678

Ellsworth ML and Pittman RN (1990) Arterioles supply oxygen to capillaries by diffusion as well as by convection. *Am J Physiol* **258**: H1240–H1243

Ellsworth ML, Pittman RN and Ellis CG (1987) Measurement of hemoglobin oxgyen saturation in capillaries. *Am J Physiol* **252**: H1031–H1040

Evans SM, Joiner B, Jenkins WT, Laughlin KM, Lord EM and Koch CJ (1995) Identification of hypoxia in cells and tissues of epigastric 9L rat glioma using EF5 [2-(2-nitro-1H-imidazol-1-yl)-N-(2,2,3,3,3-pentafluoropropyl) acetamide]. *Br J Cancer* **72**: 875–882

Falk P (1978) Patterns of vasculature in two pairs of related fibrosarcomas in the rat and their relation to tumour responses to single doses of radiation. *Eur J Cancer* **14**: 237–250

Falk P (1980) The vascular pattern of the spontaneous C3H mouse mammary carcinoma and its significance in radiation response and in hyperthermia. *Eur J Cancer* **16**: 203–217

Fenton BM and Boyce DJ (1994) Micro-regional mapping of HbO2 saturations and blood flow following nicotinamide administration. *Int J Radiat Oncol Biol Phys* **29**: 459–462

Helmlinger G, Yuan F, Dellian M and Jain RK (1997) Interstitial pH and $PO_2$ gradients in solid tumors in vivo: high-resolution measurements reveal a lack of correlation. *Nature Med* **3**: 177–182

Kennedy AS, Raleigh JA, Perez GM, Calkins DP, Thrall DE, Novotny DB and Varia MA (1997) Proliferation and hypoxia in human squamous cell carcinoma of the cervix: first report of combined immunohistochemical assays. *Int J Radiat Oncol Biol Phys* **37**: 897–905

Kerger H, Torres Filho IP, Rivas M, Winslow RM and Intaglietta M (1995) Systematic and subcutaneous microvascular oxygen tension in conscious Syrian golden hamsters. *Am J Physiol* **268**: H802–H810

Lagergren C, Lindholm A and Soderberg G (1960) Vascularization of fibromatous and fibrosarcomatous tumors. *Acta Radiol* **53**: 1–16

Lindgren AGH and Docent MD (1945) The vascular supply of tumours with special reference to the capillary angioarchekture. *Acta Pathol Microbiol Scand* **22**: 493–522

Mueller-Klieser W, Vaupel P, Manz R and Schmidseder R (1981) Intracapillary oxyhemoglobin saturation of malignant tumors in humans. *Int J Radiat Oncol Biol Phys* **7**: 1397–1404

Papenfuss D, Gross JF, Intaglietta M and Treese FA (1979) A transparent access chamber for the rat dorsal skin fold. *Microvasc Res* **18**: 311–318

Raleigh JA, Dewhirst MW and Thrall DE (1996) Measuring tumor hypoxia. *Semin Radiat Oncol* **6**: 37–45

Secomb TW, Hsu R, Dewhirst MW, Klitzman B and Gross JF (1993) Analysis of oxygen transport to tumor tissue by microvascular networks. *Int J Radiat Oncol Biol Phys* **25**: 481–489

Secomb TW, Hsu R, Ong ET, Gross JF and Dewhirst MW (1995) Analysis of the effects of oxygen supply and demand on hypoxic fraction in tumors. *Acta Oncol* **34**: 313–316

Shan SQ, Rosner GL, Braun RD, Hahn J, Pearce C and Dewhirst MW (1997) Effects of diethylamine/nitric oxide on blood perfusion in the R3230Ac mammary carcinoma. *Br J Cancer* **76**: 429–437

Smith BR, Johnson GA, Groman EV and Linney E (1994) Magnetic resonance microscopy of mouse embryos. *Proc Natl Acad Sci USA* **91**: 3530–3533

Swain DP and Pittman RN (1989) Oxygen exchange in the microcirculation of hamster retractor muscle. *Am J Physiol* **256**: H247–H255

*© Cancer Research Campaign 1999* 

Thomlinson RH and Gray LH (1955) The histological structure of some human lung cancers and the possible implications for radiotherapy. *Br J Cancer* **9**: 539–549
Torres Filho IP, Kerger H and Intaglietta M (1996) $pO_2$ measurements in arteriolar networks. *Microvasc Res* **51**: 202–212
Vinogradov SA, Lo LW, Jenkins WT, Evans SM, Koch C and Wilson DF (1996) Non-invasive imaging of the distribution of oxygen in tissue in vivo using near infra-red phosphors. *Biophys J* **70**: 1609–1617
Warren BA (1979) The vascular morphology of tumors. In *Tumor Blood Circulation*, Peterson HI (ed.) pp. 1–47. CRC Press: Boca Raton
Wilson DF and Cerniglia GJ (1992) Localization of tumors and evaluation of their state of oxygenation by phosphorescence imaging. *Cancer Res* **52**: 3988–3993

*© Cancer Research Campaign 1999*
